# A Within-Animal Comparison of Skilled Forelimb Assessments in Rats

**DOI:** 10.1371/journal.pone.0141254

**Published:** 2015-10-27

**Authors:** Andrew M. Sloan, Melyssa K. Fink, Amber J. Rodriguez, Adam M. Lovitz, Navid Khodaparast, Robert L. Rennaker, Seth A. Hays

**Affiliations:** 1 Vulintus, Inc., Dallas, Texas, United States of America; 2 University of Texas at Dallas, Texas Biomedical Device Center, Richardson, Texas, United States of America; 3 University of Texas at Dallas, Brain and Behavioral Sciences, Richardson, Texas, United States of America; 4 University of Texas Southwestern Medical Center, Neurology and Neurotherapeutics, Dallas, Texas, United States of America; 5 University of Texas at Dallas, Bioengineering, Richardson, Texas, United States of America; University of Lethbridge, CANADA

## Abstract

A variety of skilled reaching tasks have been developed to evaluate forelimb function in rodent models. The single pellet skilled reaching task and pasta matrix task have provided valuable insight into recovery of forelimb function in models of neurological injury and disease. Recently, several automated measures have been developed to reduce the cost and time burden of forelimb assessment in rodents. Here, we provide a within-subject comparison of three common forelimb assessments to allow direct evaluation of sensitivity and efficiency across tasks. Rats were trained to perform the single pellet skilled reaching task, the pasta matrix task, and the isometric pull task. Once proficient on all three tasks, rats received an ischemic lesion of motor cortex and striatum to impair use of the trained limb. On the second week post-lesion, all three tasks measured a significant deficit in forelimb function. Performance was well-correlated across tasks. By the sixth week post-lesion, only the isometric pull task measured a significant deficit in forelimb function, suggesting that this task is more sensitive to chronic impairments. The number of training days required to reach asymptotic performance was longer for the isometric pull task, but the total experimenter time required to collect and analyze data was substantially lower. These findings suggest that the isometric pull task represents an efficient, sensitive measure of forelimb function to facilitate preclinical evaluation in models of neurological injury and disease.

## Introduction

Analysis of forelimb motor function in rodents is critical to understanding motor learning, control, and recovery after neurological injury. Among the most commonly used tasks to evaluate skilled forelimb function are the single pellet skilled reaching task and pasta matrix task [1–4]. In these forelimb assessments, rodents are typically trained to reach out through an aperture, grasp a food reward, retract the limb, and eat the reward. Analysis of performance can provide endpoint metrics such as number of trials, success rate, and reaching range. Further, kinematic analysis can be performed on the complex reaching movements to provide a fine-scale description of specific motor components [1,3,4]. Both of these standard forelimb assessments have been adapted for use in rats and mice and have provided valuable insight into motor function and dysfunction after brain injury [5,6].

Recently, several automated measures of forelimb function have been developed to assay motor function in rats. Automation obviates many of the challenges motor testing, expediting and standardizing data collection and facilitating a considerably higher number of repetitions [7–9]. In some cases, automation has been applied to streamline standard tests of forelimb function, such as single pellet skilled reaching [8]. In addition to the automation of standard tasks, other novel automated operant measures of forelimb function have been developed [7,9]. One such task, the isometric pull task, is an automated task designed to quantitatively measure volitional forelimb strength and function [7]. In the isometric pull task, rats are trained to reach out through a narrow aperture in the cage, grasp a handle affixed to a force transducer, and apply force to exceed a threshold in order to trigger the delivery of a food reward. The isometric force task can be used to quantify many parameters related to forelimb function, including volitional forelimb strength, impulse, number of trials, and success rate. This task bears many similarities to the single pellet reaching and pasta matrix tasks, but differs in the metrics produced and the dissociation of target and reward characteristic of operant tasks. The development of these automated forelimb assessment tasks is relatively recent, but several studies have already employed some form of automated forelimb assessment to measure motor learning and recovery after injury [7–13].

As automated tasks become employed in more experimental paradigms, it would be useful to cross-validate these automated tasks with the well-established standard forelimb assessments. Studies rarely evaluate multiple measures of skilled forelimb function in single subjects, making it difficult to directly compare performance across tasks. In this study, subjects were trained to perform the skilled reaching task, the pasta matrix task, and the isometric pull task, in order to allow direct comparison of all three tasks in individual subjects. Performance on each task was evaluated longitudinally over the course of six weeks after ischemic lesion of the motor cortex and striatum. We report forelimb measures over time and within-subject correlations of performance on each task. Additionally, we report the time required to collect data for each task, an important consideration in reducing the time and labor requirements for forelimb assessments. We find that all tasks measure significant impairments in forelimb function on the second week after injury, but only the isometric pull task detects robust, stable deficits six weeks after lesion. Additionally, we find that the performance on all tasks is generally well-correlated after lesion. These findings demonstrate that the isometric pull task may represent an effective method to measure forelimb function in preclinical models of motor dysfunction.

## Materials and Methods

### 2.1 Subjects

Thirteen adult female Sprague-Dawley rats (Charles River; Wilmington, MA), weighing approximately 250 grams at the start of training, were used for this experiment. Rats were housed in a vivarium on a 12:12 reversed light-dark cycle with testing conducted during the dark daytime period. Subjects were food deprived during training from Monday through Friday, and were given free access to food on Saturday and Sunday. Rats were maintained at or above 85% of their initial body weight. During training, food pellet rewards were typically sufficient to maintain weight, but additional food was provided as needed. All handling, housing, behavioral testing, and surgical procedures conducted in this study conformed to NIH guidelines and were approved by the University of Texas at Dallas Institutional Animal Care and Use Committee (Protocol #14–10). All surgical procedures were performed under ketamine anesthesia, and all efforts we made to minimize suffering.

### 2.2 Common Behavioral Apparatus and Methods

**Fig 1:**
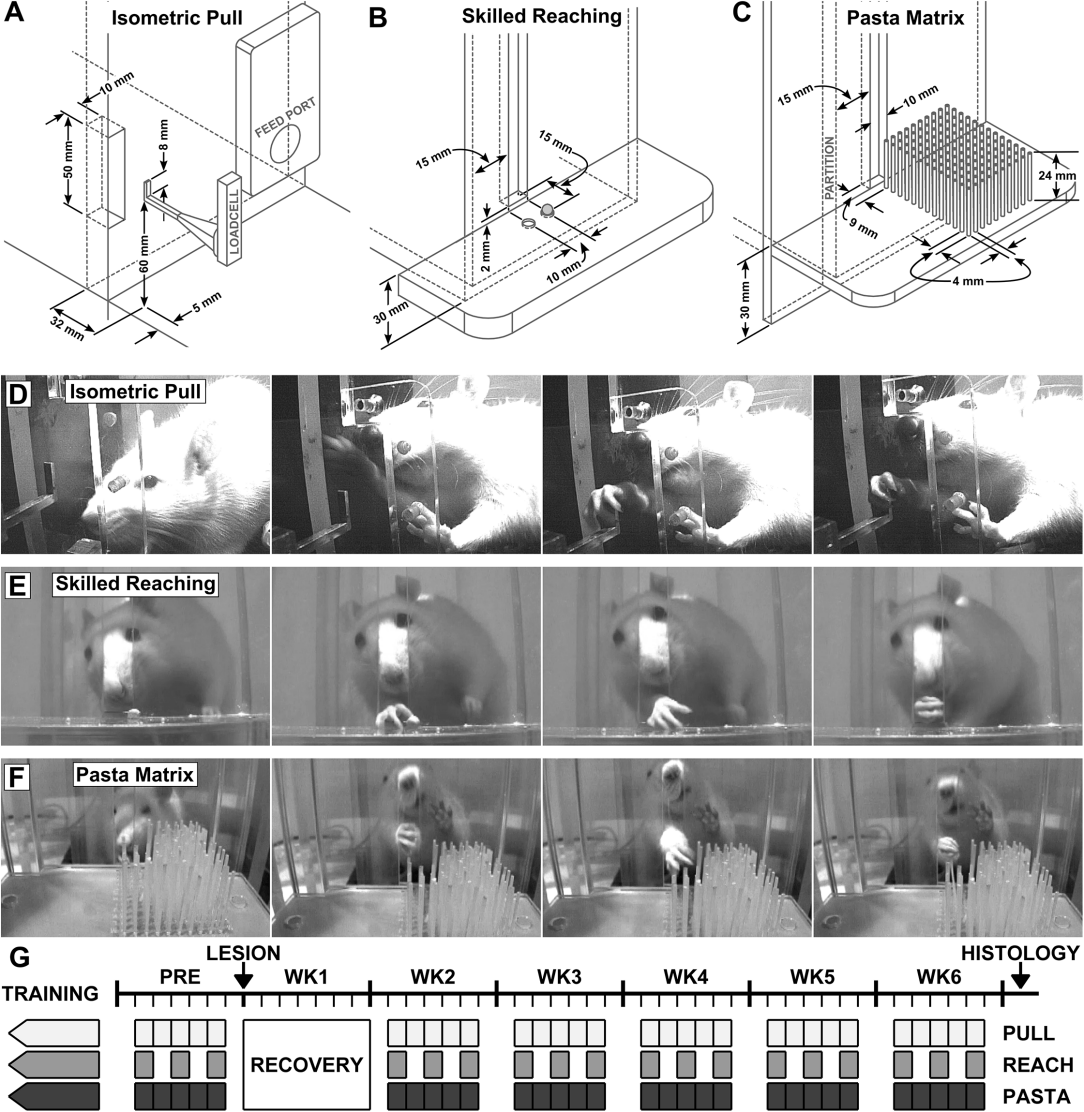
Illustrations of the behavioral apparatus are shown for the (A) Isometric Pull, (B) Skilled Reaching, and (C) Pasta Matrix tasks. Sequential images captured from videos of rats performing the tasks are shown in (D), (E), and (F), respectively. A timeline showing the task schedule prior to and following ischemic lesion is shown in (G).

The isometric pull, skilled reaching, and pasta matrix tasks were carried out in behavioral chambers with identical interior dimensions (25 x 30 x 12 cm), although separate chambers were used for each task to prevent confusion. The back wall of each chamber was customized to the requirements of the given task. Diagrams and critical dimensions for each of the tasks are illustrated in Fig 1. Training and testing on all three tasks was carried out concurrently. Once initiated, training continued on all tasks until rats performed all tasks at criterion. Subjects performed behavioral training and testing 5 days per week, Monday through Friday, while food deprived. During each day of pre- and post-lesion testing, subjects completed two 30 minute sessions on the isometric pull task and one 30 minute session on the pasta matrix task. Subjects were tested on the skilled reaching task on 3 of the 5 behavioral days per week, with each session lasting between 10 and 30 minutes, depending on the performance of the rat. Testing was performed in a random order for each task on each day to minimize circadian effects and effects of previous testing.

### 2.3 Isometric Pull Task

The isometric pull task has been previously described in detail [7]. Briefly, rats were trained to reach through a slot to grasp a stainless steel handle and to pull on the handle until they exceeded a specified force threshold in order to receive a 45 mg food pellet reward (Bio-Serv; Frenchtown, NJ). Trials began when an initiation force exceeding 10 grams was detected, after which the rats had 2 seconds within which to exceed the “hit” force threshold for that trial. If the rat did not pull with the required force within 2 seconds, the trial was scored as a “miss” and the rat as given a 2-second time-out in which no new trial could be initiated. The task was monitored through MotoTrak software (Vulintus, Inc.; Dallas, TX), which also collected and stored the force signals from each trial.

Training was carried out through several stages, beginning with a shaping stage in which the handle protruded into the chamber and any interaction with the handle resulted in a food reward. After shaping, the handle was retracted through the course of two training stages, first to 0.25 inches, recessed relative to the inner wall surface, and then to the final distance of 0.75 inches outside the cage. During these stages, the force threshold was adaptively increased by the software such that rats were required to exceed their 50th percentile force calculated over the previous 20 trials. Required force thresholds were capped at 120 grams. Force thresholds remained adaptive throughout the timecourse of the study using the same 50th percentile calculation to allow subjects with impaired pull force following ischemic lesion to remain engaged in the task. Rats were considered to be trained to criterion when the percentage of trials in which peak force exceeded 120 grams was greater than 80% over 5 consecutive training days.

### 2.4 Skilled Reaching Task

The skilled reaching task was adapted from Whishaw and Pellis [1]. Rats were trained to reach though a 1 cm wide slot to grasp and retrieve 45 mg food pellets from a tray mounted outside the cage. Food pellets were placed in two depressions in the tray, both 15 mm from the inner wall surface, and ±5 mm from the slot midline. The top surface of the tray was at a height 30 mm above the cage floor (Fig 1B). After initial training, a partition wall was placed inside the cage 15 mm to the right of the slot, and pellets were only placed in the left-most depression, relative to the rat’s position, to encourage right paw usage. Food pellets were manually placed by experimenters. Trials began when the food pellet was placed, and ended when the pellet was either successfully retrieved, dropped, or knocked away. Rats completed a total of 30 trials per testing session. Trials in which the rat successfully brought the food pellet to its mouth were scored as a “hit” and trials in which the pellet was knocked away or dropped were scored as a “miss”. Trials were automatically scored as a “miss” following 5 unsuccessful right paw attempts. Sessions were video recorded at 60 frames per second with a high-speed USB camera (Imaging Source; Charlotte, NC) for offline analysis. Testing session performance was quantified by manual scoring of the video files, and the number of reach attempts per trial with the left or right paw was also recorded.

Training on the skilled reaching task began by first placing food pellets within the slot, such that rats could reach the pellets with their mouths alone. Pellets were then gradually placed at farther distances from the inner wall surface until pellets were only present in the 15 mm depressions. After each pellet was either successfully retrieved, dropped, or knocked away, rats were trained to turn and retrieve a supplementary food pellet from an upper corner of the cage, requiring them to reset their stance prior to the next reaching attempt. After rats were trained to reset, the supplementary food pellet was only occasionally given to maintain resetting behavior. Performance for initial training sessions was manually scored online, using a tally counter to record successful reaches. Video recording and offline scoring was performed by a trained experimenter blinded to subject and began once the rat had at least 10 hits in 30 trials. Rats were considered to be trained to criterion when rats used their right paws in all attempts within a session and when mean hit rates exceeded 20% over 3 consecutive sessions.

### 2.5 Pasta Matrix Task

The pasta matrix task was adapted from Ballerman *et al*. (2001). Rats were trained to reach through a 1 cm wide slot to grasp, break, and retrieve pieces of vertically-oriented pasta (Barilla Angel Hair Pasta, Barilla America, Inc.; Bannockburn, IL) inserted into a grid-ordered field of holes. Rows of 10 pieces and columns of 13 pieces were each separated by 4mm, with the closest piece (0, 9 mm) centered with the slot 9 mm from the inner wall surface and with the field extending out and to the left to the farthest piece (40 mm, 61 mm). Pasta pieces were laser cut to 30 mm lengths and holes were 6 mm deep, leaving 24 mm protruding from the matrix plate, the top surface of which was at a height 30 mm above the cage floor (Fig 1C). A partition wall was placed inside the cage 15 mm to the right of the slot to require right-paw usage to reach the majority of the field. A total of 130 pasta pieces were available to the rat during each 30 minutes session. Performance was quantified by manual inspection of the matrix at the end of the session, counting the number of broken pasta pieces and recording their respective distances.

Training on the pasta matrix task began by introducing pasta pieces to the food-deprived rats overnight in their home cage to acclimate to the new food. In the first behavioral session on the following day, the wall of the cage was reversed such that the pasta matrix platform protruded into the cage, allowing use of both paws and the mouth, to facilitate learning of the mechanics of breaking and retrieving the pasta. On all subsequent days, the pasta matrix platform was presented opposite the slot, requiring the rat to reach through the slot to outside the cage to break and retrieve pasta pieces. Rats were considered to be trained to criterion when mean broken pasta counts exceeded 30 and had a standard deviation of less than 3 pieces over 5 consecutive days.

### 2.6 Unilateral Motor Cortex Ischemic Lesion

After reaching criterion on all three operant tasks, rats were given a unilateral cortical/subcortical ischemic lesion, similar to previous descriptions with modifications [14]. Rats were anesthetized with ketamine hydrochloride (80 mg/kg, i.p.) and xylazine (10 mg/kg, i.p.) and given supplemental doses as needed. Rats were placed in a stereotaxic frame and a craniotomy exposed primary motor cortex contralateral to the trained forelimb. Endothelin-1 (ET-1, Bachem; Torrance, CA, 1 mg/mL in saline) was injected into nine locations using a 26-gauge Hamilton syringe. The first eight injections were within the motor cortex: 2.5, 1.5, 0.5, and -0.5 AP and 2.5 and 3.5 ML from bregma, at a depth of 1.8 from the cortical surface. The ninth injection was within the dorsolateral striatum: 0 AP, 3.0 ML to bregma at a depth of 6.0 mm ventral to the skull surface. At all sites, 1.0 μL of ET-1 was injected over 2 minutes, and the syringe was left in place for 3 additional minutes. Following injections the craniotomy was sealed with a brushite-filled bone cement [15], and the skin was sutured closed. A topical antibiotic was applied to the sutured incision to prevent infection.

Following surgery, rats were returned to their home cages and allowed to recover for one week before resuming behavior. Subjects received daily subcutaneous injections of the analgesic ketoprofen (5 mg/kg) for the first 3 days, after which they were given Rimadyl tablets for the remainder of the recovery week. At the end of recovery, rats were returned to food deprivation prior to resumption of behavior.

### 2.7 Histology

Following completion of testing at the end of post-lesion week 6, all subjects were transcardially perfused with 4% paraformaldehyde. Brains were removed and sliced using a cryostat into coronal sections 40 μm thick through the extent of the lesion. Sections were stained with cresyl violet and imaged under bright field. Lesion size was measured using manual tracing on images of each section, similar to previous studies [11–13]. Histological analysis was completed with custom MATLAB software, and experimenters were blind to subject identity during manual tracing.

## Results

### 3.1 Task Acquisition

**Fig 2:**
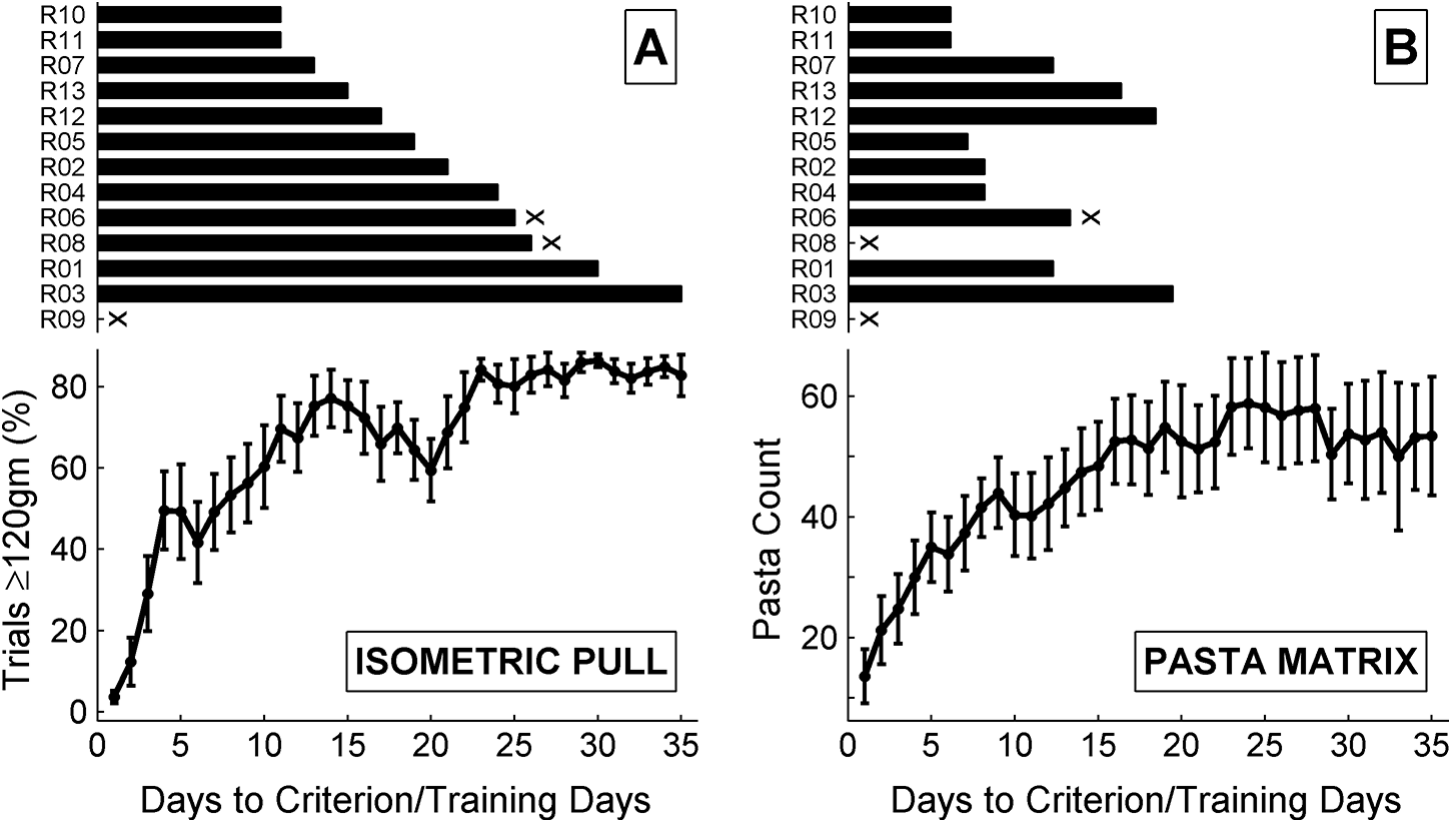
Time to criterion proficiency measured by trials with a peak force exceeding 120 gm and pasta count are shown for the isometric pull (A) and pasta matrix (B) tasks, respectively. Subjects which failed to reach criterion proficiency on all three tasks are marked with an “x”. Error bars show standard error of the mean.

Ten of the thirteen subjects that began behavioral training reached criterion proficiency on all three tasks. Acquisition measures and time to criterion proficiency for the isometric pull and pasta matrix tasks are shown in Fig 2. Of the three remaining subjects, one failed to reach criterion on all three tasks, one failed to reach criterion on the skilled reaching task and the pasta matrix task, and one failed to reach criterion on only the skilled reaching task. Failure to meet criterion on the isometric pull and pasta matrix tasks was due primarily to subjects failing to exceed required hit rate and pasta count thresholds, respectively. Failure to meet criterion on the skilled reaching task was primarily due to persistent left paw usage.

### 3.2 Pre-Lesion Task Performance

Baseline performance on each of the three tasks was measured for the 10 trained subjects over one week prior to the lesion. Subjects’ baseline hit rates, the percentage of trials in which force exceeded 120 gm, for the isometric pull task ranged from 80.3% to 96.5% (mean = 88.7% ±1.9%), with mean peak forces ranging from 136 gm to 198 gm (mean = 151 ±6 gm). Baseline hit rates for the skilled reaching task ranged from 20.0% to 50.0% (mean = 33.3% ±3.0%). Baseline pasta counts for the pasta matrix task ranged from 36.1 to 82.0 (mean = 60.5 ±5.1), with mean farthest reach distances ranging from 33.6 mm to 55.7 mm (mean = 44.9 ±2.3 mm). To evaluate the consistency of pre-lesion performance across subjects, coefficient of variation was calculated for each task. Baseline hit rates from the isometric pull task had significantly less dispersion, measured by the coefficient of variation, than hit rates from the skilled reaching task (Isometric Pull: 0.064; Skilled Reaching: 0.268; F-Test, F[9,9] = 17.34, p < 0.001) or pasta counts from the pasta matrix task (Pasta Matrix: 0.255; F-Test v. isometric pull, F[9,9] = 15.70, p < 0.001,). The dispersion of baseline skilled reaching hit rate and pasta matrix counts were not significantly different (Skilled Reaching v. Pasta Matrix; F-Test, F[9,9] = 0.91, p = 0.884). Together, these findings indicate that the skilled reaching and pasta matrix tasks had greater pre-lesion performance variability between subjects compared to the isometric pull task

Task performance, relative to the group mean, on one task did not generalize to other tasks. Hit rate for the isometric pull task, hit rate for the skilled reaching task, and retrieved pasta count for the pasta matrix were not significantly correlated (Spearman rank correlation, α = 0.05). No rat had the best performance on more than one task, and similarly no rat had the worst performance on more than one task.

### 3.3 Task Performance following Contralateral Motor Cortex Lesion

**Fig 3:**
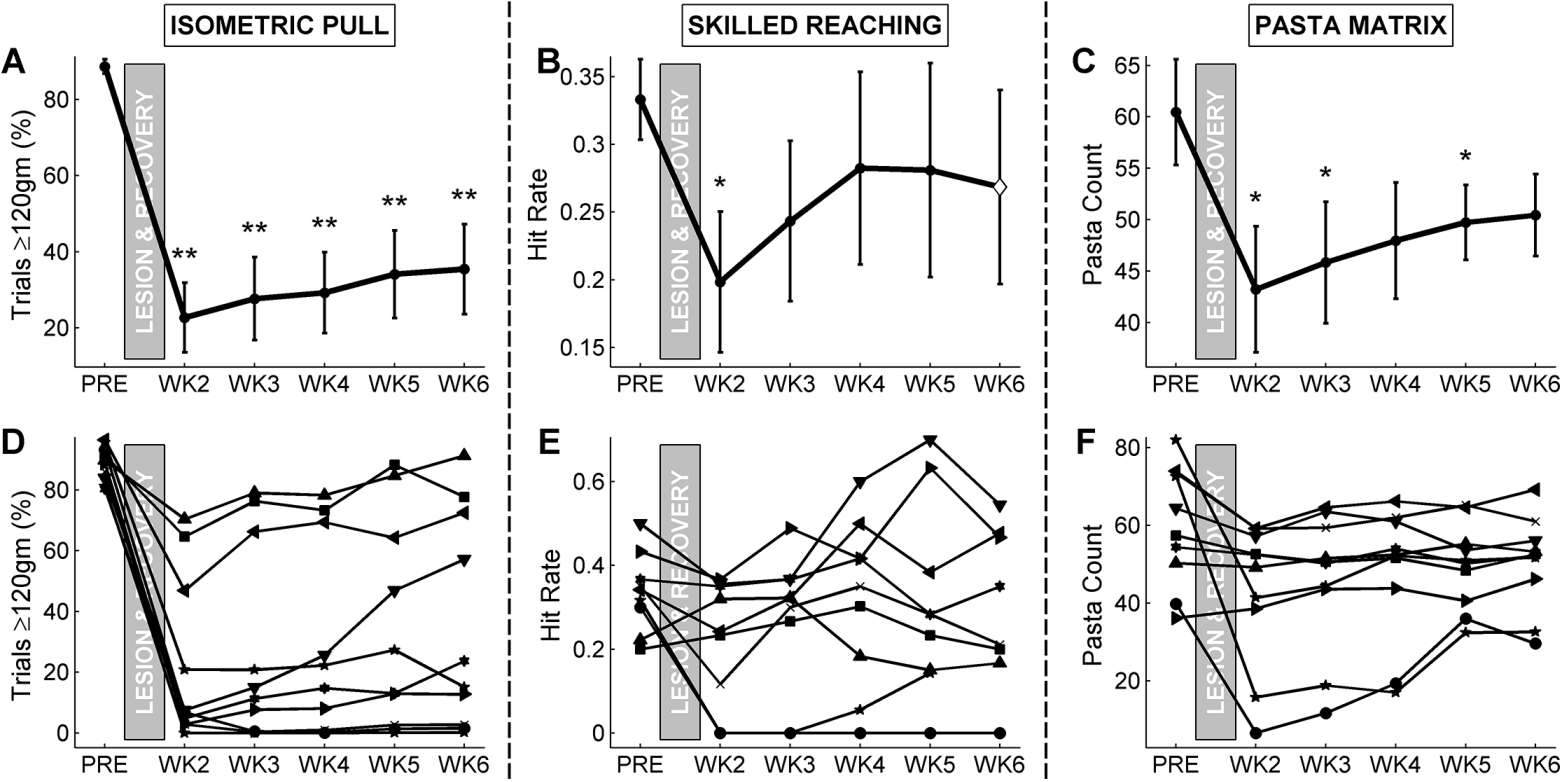
Primary performance measures of the percent of trials with a peak force exceeding 120 gm for the isometric pull task (A), hit rate for skilled reaching task (B), and pasta count for the pasta matrix task (C) are shown from 1 week prior to 6 weeks following unilateral motor cortex lesion. Error bars show standard error of the mean. Significant difference from pre-lesion performance are indicated with asterisks (paired t-test; *, *p* < 0.05; **, *p* < 0.001). One point for which only 9 subjects’ measures could be collected is marked with a ◊. Individual subjects’ performance on each task is shown in (D), (E), and (F), respectively.

Once trained to proficiency on all tasks, subjects received a unilateral ischemic lesion of motor cortex and striatum. Behavioral testing resumed one week after lesion. Ischemic lesion significantly worsened performance relative to pre-lesion values on all tasks (Pre-lesion v. Week 2 post-lesion, paired t-test; isometric pull hit rate, *p* < 0.001; skilled reaching hit rate, *p* = 0.019; pasta matrix count, *p* < 0.022). Post-lesion primary measures of isometric pull hit rate, skilled reaching hit rate, and pasta matrix count are shown in Fig 3.

Ischemic lesion produced a wide range of impairments across the three tasks. Hit rates on the isometric force task for all 10 subjects during post-lesion week 2 were lower than pre-lesion baselines, with a minimum deficit of 21.6% and maximum deficit of 100%, relative to baseline. Week 2 hit rates on the skilled reaching task were lower than pre-lesion baseline for 8 of 10 subjects, with a minimum deficit of 4.5% and a maximum deficit of 100%, relative to baseline. Week 2 pasta counts from the pasta matrix task were lower than pre-lesion baseline for 9 of 10 subjects, with a minimum deficit of 2.2% and a maximum deficit of 83.4%, relative to baseline. All three tasks measured a significant impairment in forelimb function on the second week post-lesion.

### 3.4 Task Performance Recovery during Post-Lesion Testing

Testing on all three behavioral tasks continued for a total of five weeks. While performance deficits were seen on all tasks during the first week of behavior following lesion, a lasting deficit was only significant for the isometric pull task through all 5 weeks of post-lesion behavior. Isometric pull hit rate was significantly decreased relative to pre-lesion baseline across all post-lesion weeks (Pre-lesion v. each week, paired t-test; all *p* < 0.001). Skilled reaching hit rate deficits, by contrast, were not significant following post-lesion week 2 (α = 0.05). Pasta matrix count deficits were significant for some, but not all, weeks (2, 3, and 5, p < 0.05 for each; 4 and 6, p > 0.05).

### 3.5 Task Impairment Correlations

**Fig 4:**
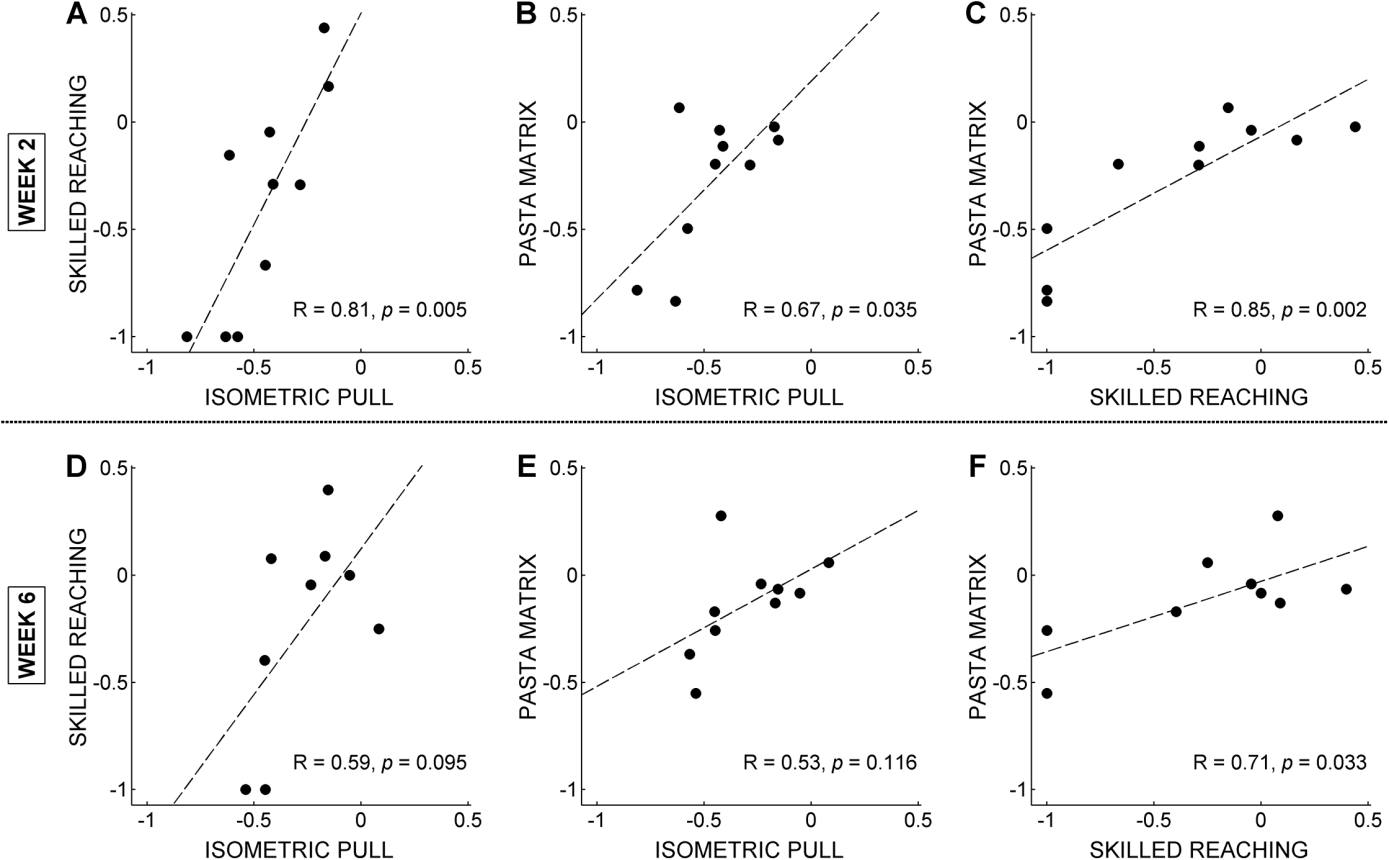
Normalized peak force per trial, relative to subjects’ pre-lesion baselines, for the isometric pull task is plotted against normalized hit rate for the skilled reaching task for post-lesion weeks 2 (A) and 6 (D) and plotted against pasta counts for the pasta matrix task for weeks 2 (B) and 6 (E). Skilled reaching normalized hit rates are plotted against normalized pasta matrix counts for weeks 2 (C) and 6 (F). Dotted trendlines were fitted by linear regression and *R* and *p* values were calculated using Spearman’s rank correlation.

The degree of subjects’ motor impairment following the unilateral ischemic lesion was generally consistent across tasks (Fig 4). Performance deficits are significantly correlated between all three tasks in week 2 (Spearman’s rank correlation, α = 0.05). However, only normalized skilled reaching hit rates and pasta matrix counts are significantly correlated in week 6 (*p* = 0.033). The lack of a correlation between the isometric force task and either the pasta matrix task or skilled reaching task at week 6 appears to arise from the difference between the large effect size for the isometric force task and the smaller effect size for the other tasks.

### 3.6 Secondary Performance Measures

**Fig 5:**
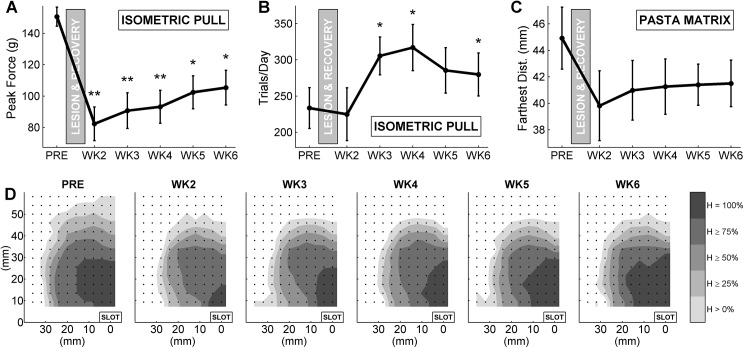
A) Mean peak force per trial (A) and number of trials per day (B) are shown for the isometric pull task (A). Distance to the farthest broken pasta (C) and iso-value contours of hit rate for individual pasta pieces (D) area shown for the pasta matrix task. Error bars show standard error of the mean. Significant difference from pre-lesion performance is indicated with asterisks (paired t-test; *, *p* < 0.05; **, *p* < 0.001).

The isometric pull and pasta matrix tasks provide secondary performance measures which do not require additional post-hoc video analysis. These secondary measures are separate from, but related to, primary measures of isometric pull hit rate and pasta matrix count and can be used for further behavior analysis. Post-lesion deficits on secondary performance measures for each of the three tasks were generally consistent with deficits seen on primary (Fig 5).

Mean peak force per trial for the isometric pull task was significantly decreased relative to pre-lesion baseline for all post-lesion weeks (Pre-lesion v. each week, paired t-test, α = 0.05). Despite remaining significantly reduced compared to pre-lesion, peak force did show some recovery over the 5 post-lesion weeks (2-way ANOVA, main effects of subject and time; F_time_(4,36) = 12.02, *p*_time_ < 0.001). Daily trial count for the isometric pull task was also significantly increased for weeks 3, 4, and 6 (paired t-test, α = 0.05), suggesting that subjects may perform more trials to compensate for lower hit rates.

**Fig 6:**
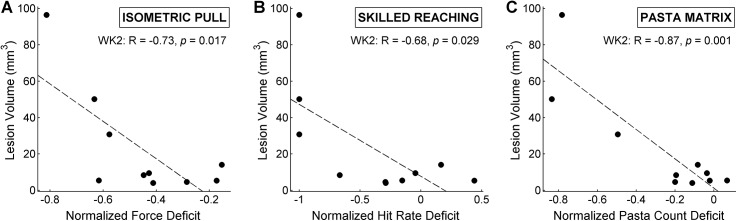
Total lesion volume is plotted against post-lesion Week 2 normalized peak force deficit for the isometric pull task (A), normalized hit rate deficit for the skilled reaching task (B), and normalized pasta count deficit for the pasta matrix task (C). All deficit values are normalized to individual subjects’ pre-lesion baselines. Dotted trendlines were fitted by linear regression and *R* and *p* values were calculated using Spearman’s rank correlation.

Analysis of broken pasta in the pasta matrix tasks also provided an estimate of the extent of spatial reach for each subject. Farthest reach distance, measured from the center of the slot to the farthest broken pasta piece, was decreased in all post-lesion weeks, but not significantly (paired t-test, α = 0.05). Contour plots of mean hit rate for individual pasta pieces (Fig 6D) show decreases in the overall spatial extent of reach, but centroid coordinates are not significantly changed in either the x- or y-direction (paired t-test, α = 0.05).

### 3.7 Histology

Endothelin-1 injections produced a range of lesion volumes, from a minimum of 4.0 mm^3^ to a maximum of 96.5 mm^3^ (mean = 22.8 ±9.9 mm^3^). Lesion volumes were significantly correlated with performance measures on all three tasks (Spearman rank correlation, α = 0.05) during post-lesion week 2 testing (Fig 6). Note that for the skilled reaching task, subjects with the 3 largest lesions were unable to score a single hit during post-lesion week 2, suggesting that the task may not have appropriate range for large lesion models. All subjects, regardless of lesion size, maintained non-zero post-lesion performance on the isometric pull and pasta matrix tasks, although some subjects did not recover the ability to exceed 120 gm of force on the isometric pull task.

### 3.8 Experimenter Time Requirements

A major motivation in development of new motor assessments is the reduction of required experimenter supervision and manual scoring time investments. Throughout the course of the study, an accounting was kept of time spent by experimenters conducting or analyzing each motor assessment, and a breakdown of time requirements for each task is shown in Table 1. The isometric force task required the least experimenter time, approximately 1 minute per session, to transfer rats to and from the behavioral cage and to initiate the session in the software. The pasta matrix task required the next least amount of experimenter time, approximately 16 minutes per session, primarily spent resetting pasta in the matrix prior to the session and visually inspecting and recording broken pieces following the session. The skilled reaching task was the most time-intensive for experimenters, requiring approximately 40 to 60 minutes per session, spent primarily in directly supervising and participating in the behavior and in analyzing session videos offline. In total, summing across all subjects and all training, pre-lesion, and post-lesion testing sessions, 16 hours of experimenter time was required to collect isometric pull task data for this study, versus 188 hours for skilled reaching and 110 hours for pasta matrix.

**Table 1:** Experimenter Supervision and Scoring Time Requirements.

Task	Pre-Session Preparation	Pre-Session Handling	Direct Session Supervision	Post-Session Handling	Offline Scoring	Total Time Per Session	Total Pre-Criterion Training Time	Total Post-Criterion Testing Time
Isometric Pull	none		none		none	1 minute	6 hours	10 hours
Skilled Reaching	none	30 seconds (transfer to behavioral cage)	10–30 minutes	30 seconds (transfer to behavioral cage)	30 minutes (video scoring)	42–62 minutes	47 hours	141 hours
Pasta Matrix	10 minutes (matrix reset)		none		5 minutes (matrix scoring)	16 minutes	30 hours	80 hours

Despite requiring the least amount of experimenter time, the isometric pull task also yielded a very large number of total trials per subject (8033 ±727), not including training, whereas the skilled reaching task only yielded 370 ±34 total pre- and post-lesion trials. Trial counts for the pasta matrix task are difficult to define, since the retrieval difficulty of each piece differs, but subjects retrieved, on average, 1488 ±136 of a total of 3900 available pasta pieces during pre- and post-lesion testing. These trial counts, and the time required to collect them, highlight a key advantage of automated behavioral tasks like the isometric pull task.

## Discussion

In this study, we compared forelimb motor function after ischemic lesion in subjects trained on three preclinical measures: the skilled reaching task, the pasta matrix task, and the isometric pull task. All three tasks measured a significant impairment in forelimb performance on the second week after lesion. By the sixth week post-lesion, only the isometric pull task measured a significant impairment in forelimb function. Normalized performance on all tasks is well-correlated on the second week after lesion but generally less correlated on the sixth week after lesion. Comparison of the time required by experimenters to set up and analyze each task suggests that the isometric pull task has advantages in efficiency and dataset size compared to the other tasks.

Longitudinal measurement of performance deficits is a key consideration for studies of motor recovery. Following ischemic lesion of the motor cortex and striatum, performance on all three tasks is significantly impaired. Consistent with previous studies, we find that the isometric pull task can measure long-term impairments in forelimb function despite extensive post-lesion training [10–12]. Both the pasta matrix task and the skilled reaching task initially detect significant impairments in function following lesion. However, these effects are transient, and behavioral measures fail to detect a significant reduction in forelimb function after several weeks.

Task-oriented rehabilitative training is regarded as one of the more effective rehabilitative interventions after brain injury [16]. The long-lasting reduction in forelimb performance on the isometric pull task is observed in spite of extensive task-focused training after lesion. This may model the chronic functional deficits in upper limb use after brain injury in patients. The absence of lasting detectable impairments on the skilled reaching and pasta matrix tasks in the current study may be due to the extensive degree of training that the subjects received on all tasks following injury. However, isometric pull task yields the largest observable deficits in spite of more extensive training compared to the other two tasks, suggesting that it may be more sensitive. Without regard for specific metrics of interest (i.e., pasta matrix for reaching distance), the larger effect size on the primary measure of performance provided by the isometric pull task indicates that this task may be more optimal for long-term studies.

It’s important to note that the apparent recovery of pasta matrix and skilled reaching performance is based on end-point measures, which can reflect both true recovery and behavioral compensation [17,18]. Kinematic analysis of forelimb movement may reveal lasting forelimb dysfunction on all tasks that is not observed in the performance measures reported in this study. Kinematic analysis of rodent reaching behavior; however, has not historically been amenable to high-throughput analysis, although several semiautomated methods have been developed [19,20]. While compensation is likely reflected in measures of forelimb performance in the tasks employed in this study, the larger effect size seen with the isometric pull task suggests that the task may reduce compensation and better reveal true deficits in performance.

In addition to effect size, the time required to collect data is a key consideration in behavioral testing. Consistent with previous studies, subjects took more days to reach asymptotic proficiency on the isometric pull task pull task compared to the pasta matrix or skilled reaching tasks [7,21]. As all the tasks require similar reach and grasp movements, it is likely that training on multiple tasks interacts and may alter the length of training time compared to studies in which subjects are trained on only one task. Once subjects reached proficiency, the total time burden on the experimenter was significantly lower for isometric pull compared to the other tasks over the course of the study. This is due to substantially shorter hands-on time required to conduct the experiment and analyze data. The rapid collection and analysis of data, in conjunction with the substantially greater number of trials, indicates that the isometric pull task is the most efficient task employed in this study.

A major advantage of the isometric pull task is the high degree of automation. The task is largely software controlled, which minimizes user error and facilitates rapid, accurate data collection. Additionally, the difficulty of the task can be controlled by adjusting rewards thresholds either manually or automatically in the software during a session. Automatic scaling of reward threshold, or adaptive thresholding, was used in this study to set threshold at the median force of the preceding twenty trials. Adaptive thresholding has the potential to accelerate training time. Previous studies utilizing the isometric pull task performed shaping and training in stages using fixed (non-adaptive) success thresholds. The adaptive threshold used in this study reduced the training time to ~21 days compared to ~23–26 days with fixed thresholds [7,10,12]. Without interleaved controls, it is not possible to make direct comparisons between fixed and adaptive threshold training; however, a cursory comparison would suggest that an adaptive threshold may promote faster task acquisition and performance gains than a fixed threshold. Additionally, adaptive thresholds may allow flexibility to apply the task to injury models with varying degrees of dysfunction.

One limitation of the present study is the absence of paw preference selection. While many studies define a dominant paw prior to lesion, the present study exclusively restricted use to the right paw for all subjects on all tasks. Selective lesioning of the dominant paw may produce larger behavioral impairments, but the magnitude in impairment is likely to be consistent across all tasks. Additionally, to allow comparison of standardized performance across tasks, analysis was largely restricted to the primary measure of performance on each task. Many skilled reaching studies have used slow-motion kinematic analysis in conjunction with primary measures [1,18]. These finer metrics of forelimb motion allow observation of more subtle, lasting deficits in forelimb performance which may persist longer than reductions in endpoint performance measures, but require substantially more analysis time. While not performed in the present study or previous studies, the isometric pull task is amenable to kinematic analysis, which would provide greater richness to forelimb analysis using this task.

## Conclusions

Forelimb assessments are critical to understanding motor learning, control, and recovery. Rigorous longitudinal behavioral experiments are time- and labor-intensive, thus it is necessary to select behavioral tasks that best evaluate the metric of interest in order to reduce the cost and number of subjects required to complete a study. This study provides a within-subject comparison of performance on several common skilled forelimb assessments in rats to allow direct comparison across tasks. Standard measures of performance on all tasks reveal deficits in motor function on the second week after ischemic lesion of the motor cortex and striatum. Lasting deficits in performance are only observed on the isometric pull task. Additionally, the time required to conduct and analyze the isometric pull task is shorter than the other tasks. These findings indicate that the isometric pull task represents an efficient, flexible method to assay skilled forelimb function in rats.
